# Characterization of animal tissues for Monte Carlo simulations in ion beam therapy

**DOI:** 10.1002/acm2.70836

**Published:** 2026-10-05

**Authors:** Liu Xiaoli, Michael F. Moyers

**Affiliations:** ^1^ Department of Medical Physics Shanghai Proton and Heavy Ion Center Shanghai China; ^2^ Shanghai Key Laboratory of Radiation Oncology Shanghai China; ^3^ Shanghai Engineering Research Center of Proton and Heavy Ion Radiation Therapy Shanghai China

**Keywords:** carbon ions, CT number, dual energy CT index, elemental composition, protons, relative linear stopping power

## Abstract

**Purpose:**

To obtain better elemental compositions (ECs), physical densities (PDs), and relative linear stopping powers (RLSPs) of tissues scanned by single or dual energy computed tomography for use by Monte Carlo (MC) simulations of light ion beam therapy.

**Methods and materials:**

The RLSPs of 12 animal tissue samples were measured using several different energy proton and carbon ion beams. The EC of each sample was analyzed using the following modern laboratory techniques: Inductively coupled plasma optical emission spectrometry (ICP‐OES), inductively coupled plasma mass spectrometry (ICP‐MS), organic elemental analysis (OEA), and ion chromatography (IC). RLSPs were calculated from both measured ECs and literature ECs and then compared with measured RLSPs. All samples underwent single‐energy CT (SECT) and dual‐energy CT (DECT) scanning after which a dual‐energy index (DEI) was derived. A mapping from computed tomography number (CTN) and DEI to PD was performed.

**Results:**

For each tissue sample, the measured RLSPs showed no significant dependence on beam energy or ion species, with all values agreeing to within ± 1% except for lung (within ± 2%). RLSPs directly measured and those calculated using measured PDs and ECs agreed within ± 2% except for lung. Measured RLSPs were slightly closer to values from laboratory‐determined ECs than to literature ECs.

**Conclusions:**

RLSP measurements were independent of beam energy and ion species. The main determinants of accurate CTN to RLSP conversion are the PDs and fractional hydrogen content of the tissues. The accuracy of PD and EC for input into Monte Carlo transport calculations can be improved by combining DEI and scaled CTN.

## 1 INTRODUCTION

The steep dose gradient of proton and carbon ion beams makes ion beam therapy highly sensitive to uncertainties in CTN accuracy, conversion to RLSP, patient alignment, and motion.^1^, ^2^, ^3^, ^4^, ^5^ Refining the materials in a treatment planning system (TPS) database is therefore essential to performing accurate penetration calculations. In most TPSs for charged particles, penetration is determined by first converting each voxel's Hounsfield unit (HU) to an RLSP value and then integrating across the voxels in the beam path. The conversion to RLSP typically uses one of three approaches: Direct measurement with tissue substitutes,^6^ stoichiometric calibration,^7^, ^8^ or a simplified method using air, water, and a high‐density material.^9^ Numerous studies have reported HU‐RLSP relationships for inanimate materials, and a few for real animal soft tissues.^10^, ^11^, ^12^, ^13^, ^14^ On the other hand, very few studies have included high‐density bone, and none have reported data for teeth, likely due to experimental difficulties.

MC simulations are considered more accurate than traditional algorithms for dose predictions because they model ion scattering and nuclear interactions on an event‐by‐event basis, rather than relying solely on RLSP to calculate penetration and dose deposition. To achieve this accuracy, they require both EC and PD for each voxel in a patient image. Portal optimization, however, still needs RLSP to determine the required penetration for target coverage, normal tissue protection, and accelerator energy selection. One problem with SECT is that some materials with the same SCTN may have different RLSPs, PDs, and ECs. DECT provides additional information from which those properties may be determined on a voxel‐by‐voxel level thus better supporting MC algorithms.^15^, ^16^ Additionally, DECT scans can reduce artifacts due to beam hardening and implanted materials thereby improving image quality compared to SECT which may lead to better structure segmentation and further enhancement of material characterization for MC calculations.^17^ Although DECT was proposed in 1976, its application to ion beam therapy is recent.^18^ Moreover, most tabulations of tissue ECs were published more than 30 years ago,^19^, ^20^, ^21^ before modern analytical tools became available. Recent advances offer improved precision: routine ICP‐OES achieves ≤ 2% relative standard deviation (RSD), with high‐performance methods reaching < 0.2% relative uncertainty.^22^ ICP‐MS can reach < 5% RSD for most elements in soft tissues.^23^ According to the instrument manufacturer, CHNS‐O can provide absolute standard deviations ≤ 0.1% for carbon (C), hydrogen (H), nitrogen (N) and sulfur (S).^24^ Comprehensive reviews of EC validation parameters are available.^25^

The goal of this study was to determine EC using modern elemental analysis and to combine these data with independently measured PD to refine the conversion of SECT and DECT numbers into EC and PD for MC calculations. Fresh animal samples and some inanimate materials were first scanned with SECT and DECT. Their RLSPs were then measured using several proton and carbon ion beam energies. After these radiological measurements, the samples were sent to a laboratory for EC analysis. Both measured and literature ECs were then used to calculate RLSPs and compare them with the measured values. The calculation method was cross‐checked using six GAMMEX 467 inserts, with results compared to their previously measured RLSPs.

## 2 METHODS AND MATERIALS

### 2.1 Sample preparation

Elemental and radiological measurements were performed on twelve fresh tissue samples from pigs (lung, adipose, kidney, liver, hind leg muscle, heart, brain, cartilage, neck scapula) and cows (vitreous humor, mandibular ramus, and molar teeth). To restrict measurements only to those tissues of interest, during sample preparation the kidney, heart, and liver samples were meticulously cleaned to remove fascia, adipose, and vascular tissue. Soft tissue surrounding bone and teeth was similarly removed. The entire pig brain was used without separating gray matter, white matter, or ventricles. For each sample, the PD was determined by measuring its weight and its volume. For weight measurements, before placing a sample into its container, the empty container weight was measured with a precision electronic scale. After inserting the sample, the container was weighed again; the difference gave the sample weight. For the volume measurement, a water displacement method was used. A 64 mm diameter beaker was partially filled with water (for tooth and cartilage samples, a 54 mm diameter beaker was used). The water height was recorded using a vernier caliper. The sample was then submerged and the new water height was recorded. The change in water height was converted to a change in volume and the density was calculated by dividing the sample weight by the change in volume. For low‐density tissues that tend to float, a thin metal rod was used to gently hold the sample below the water surface. For the vitreous humor sample, the volume was determined by direct calculation from the internal dimensions (length, width, and height) of the container.

For CT scanning and RLSP measurements, most samples were placed in polymethylmethacrylate (PMMA) containers with inner dimensions of 80 mm  ×  80 mm  ×  25 mm; cartilage, vitreous humor, and teeth were placed in 30 mm  ×  30 mm  ×  30 mm containers. To prevent internal displacement during transport and between measurement sessions, soft tissues were kept intact and gently packed into the containers without air gaps; bone samples were additionally secured laterally with shaped supports or foam inserts. The containers were stored and transported in a fixed orientation to avoid shifting, and the upper openings were sealed with plastic wrap to maintain sample stability. Each container had scribed crosses on the proximal and distal surfaces and horizontal lines on the lateral surfaces to assist with aligning the samples with the ion beam using the treatment room lasers. The left panel of Figure 1 shows example samples inside the containers. Due to their length, cartilage and teeth samples were cut and stacked inside the containers. The mandible and scapula samples were difficult to cut and compress so they were fixed securely within the containers with the beam passing through a region of homogeneous physical thickness. For cartilage, tooth, mandible, and scapula, the integrated depth‐ionization distribution was measured with the surrounding space filled with water. Consequently, for the RLSP measurements of these samples (Section 2.3), the shifted beam range therefore included contributions from the container, some water, and the tissue. The physical thickness of each tissue sample actually traversed by a single narrow ion beam spot was determined by viewing CT images on a TPS and using the distance measurement tools to measure the thickness of each component in the axial CT plane coaxially with the spot beam direction. The right panel of Figure 1 shows the CT images and beam paths for the samples displayed in the left panel.

**FIGURE 1:**
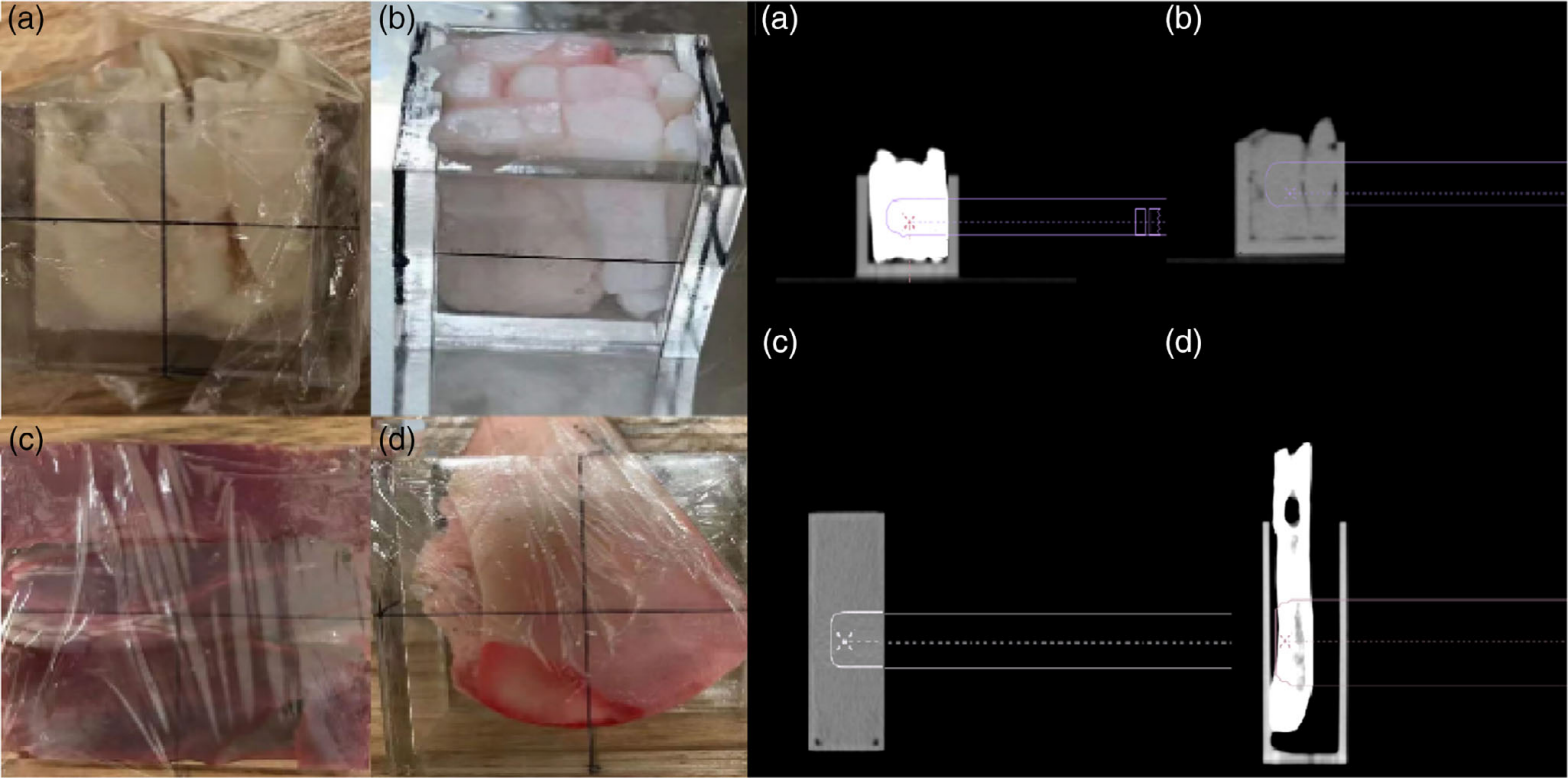
Photographs of sample containers (left) and corresponding CT images with beam paths (right) for: (a) teeth, (b) cartilage, (c) heart, (d) mandible.

To minimize tissue degradation, all CT measurements and RLSP measurements were performed within 12 h after sample preparation. Samples were kept refrigerated except during measurement.

### 2.2 CT scanning

A 64‐detector row CT scanner (SOMATOM Definition AS, Siemens Healthcare, Germany) was used for all CT scans in helical mode with a 500 mm scan field of view, pitch of 0.55, and a 16 × 1.2 mm acquisition. The reconstructed images had a center‐to‐center slice interval of 1.5 mm and a slice width (thickness) of 1.0 mm. This unit is often called a “CT on rails” because the gantry moves while the patient positioner top remains stationary. For single‐energy scans, the tube voltage was 120 kVp and the tube current was 300 mAs. For dual‐energy scans, because only a single x‐ray tube was available, the first scan was acquired at 80 kVp followed by a second scan at 140 kVp. The single‐ and dual‐energy scans were performed consecutively without moving the tissue sample. For each sample, CT images were reviewed on a Syngo TPS (Siemens, Erlangen, Germany). For each sample, a homogeneous CT number region suitable for RLSP measurement was identified, and the ion beam path is shown in the right panel of Figure 1.

The value of a voxel in a CT image is often expressed in HU. For this study, however, voxel values were expressed simply as a CTN^,^ ^26^ given by Equation (1):

(1) $$CTN=\left(\frac{\mu_{m}}{\mu_{water}}\right)\;\times \;1000$$

where $\mu_{m}$ and $\mu_{water}$ are the x‐ray linear attenuation coefficients of the material and of water, respectively, for an effective beam energy. The use of CTN instead of HU was chosen because the relationships between CTN, physical density, and RLSP are more intuitive, the analyses are simplified, and consistency is maintained with earlier literature.^27^

The average CTNs and standard deviations for each sample were then measured over three 10 mm diameter spherical regions‐of‐interest (ROIs) coinciding with the selected beam axis. To reduce the influence of fluctuating CT calibrations, sample sizes, and artifacts, the single‐energy CTNs were scaled according to Equation (2) given by Wrightstone et al.^27^ and reproduced in Moyers et al.:^11^

(2) $$SCTN=\left(\frac{CTN_{m}-CTN_{water}}{CTN_{water}-CTN_{air}}+1\right)\;\times \;1000$$

where $CTN_{m}$, $CTN_{water}$, and $CTN_{air}$ are the single‐energy CTNs of the material, water, and air, respectively.

For DECT scans, a dual‐energy index (DEI) was determined for each voxel using Equation (3), which is based on Equation (1) in Krauss et al.^28^ but transformed to use SCTN to obtain accurate DEIs for low‐density materials:

(3) $$\mathrm{DEI}=\frac{\mathrm{SCT}N_{\mathrm{low}}-\mathrm{SCT}N_{\mathrm{high}}}{\mathrm{SCT}N_{\mathrm{low}}+\mathrm{SCT}N_{\mathrm{high}}}$$

where SCTN_low_ and SCTN_high_ represent the SCTNs of the image pixels at the low and high kVps respectively.

### 2.3 RLSP measurements

RLSPs were measured at three energies for each of two ion species: Proton and carbon ion beams. As mentioned in Section 2.1, RLSPs were measured for single narrow ion beam spots using a Peakfinder® device (PTW, Germany). The device consists of a pair of parallel‐plate ion chambers with a variable‐thickness water column positioned between them. By incrementally varying the thickness of the water column and recording the ionization at each step, a laterally integrated depth‐ionization distribution was measured. Typically, 100 to 200 steps were used (depending upon the beam energy) although only a few steps are actually required around the peak and within the distal edge of the distribution. From reference and test sample measurements, the RLSP can be determined. The diameter of the parallel‐plate ion chambers was approximately 82 mm, much larger than the beam spot full‐width at half‐maximum (FWHM) diameters (3.4 to 15.1 mm at isocenter). For this beamline, the virtual source‐to‐isocenter distance for the scanning magnets is approximately 7.5 m and the vacuum window to isocenter is approximately 1.4 m. Although the beam spot is focused at the isocenter, scattering from the vacuum window, beam monitor chambers, and air predominate the spot size at the isocenter making it worthwhile to move the water column upstream as much as possible to achieve a small spot size. A photograph of the Peakfinder configured for RLSP measurements close to the exit of the radiation head is shown in Figure 2. Table 1 provides selected beam characteristics.

**FIGURE 2:**
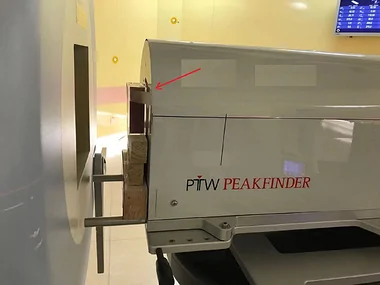
The setup for RLSP measurements using the Peakfinder consists of the beam exiting the radiation head on the left, then passing through the tissue sample and container (red arrow), and finally entering the Peakfinder device.

**TABLE 1:** Characteristics of the proton and carbon ion beams utilized for the experiments in this study.

Characteristic	proton			carbon ion		
Nominal ion energy per nucleon (MeV/n)	104.5	150.1	221.1	195.7	285.4	430.1
FWHM at the isocenter in air (mm)	15.1	11.2	8.1	5.2	4.0	3.4
Depth of the peak in water (mm)	84.1	159.2	310.7	84.2	159.3	310.5

Before measuring a single‐spot integrated depth‐dose distribution behind a tissue sample, a reference distribution was acquired using a water‐filled container. The water thickness at the distal 90% dose deposition point (R_90,w_) was recorded. The water‐filled container was then replaced with one containing the animal tissue, and the Peakfinder scan was repeated to obtain the corresponding water thickness for the material (R_90,m_). The measured RLSP for each tissue sample was then calculated using Equation 5:

(4) $$RLSP=\left(\frac{R_{90,w}-R_{90,m}}{t_{m}}\right)+1$$

where $t_{m}$ is the thickness of the tissue sample along the beam path. Both proton and carbon ion beams were measured without repositioning the tissue samples.

### 2.4 Elemental analysis

After the radiological measurements were completed, the samples were sent to a local testing laboratory for elemental analysis. Elemental composition was determined using four methods. OEA, also known as CHNS‐O, combusts milligram‐sized samples in pure oxygen; the resulting gases are separated by gas chromatography and quantified by thermal conductivity, yielding mass fractions of C, H, N, S, and O. ICP‐OES digests the sample into a liquid, nebulizes it into an argon plasma, and measures the atomic emission spectra to quantify less abundant elements: Na, Mg, P, K, Ca, Fe, Si, and Cu. ICP‐MS follows the same sample introduction as ICP‐OES but detects ions by mass spectrometry, achieving trace‐level sensitivity on the order of parts per 10^12^ for the same elements. Ion chromatography (IC) separates anions based on their affinity to an ion‐exchange resin; the eluted ions are detected by conductivity, providing concentrations of Cl^−^, I^−^, F^−^, and SO_4_^2^^−^.

### 2.5 RLSP calculations and comparisons

Three sets of RLSPs were calculated for each sample material. The first set used the ECs determined by the laboratory and the measured physical density. The second set used the ECs and PDs taken from the literature.^20^ The third set used the literature ECs combined with the measured PDs. For all sets, the ECs were input into the program LET ^29^ (Brookhaven National Laboratory, New York) to calculate the RLSPs. The percentage difference between calculated and measured RLSPs was evaluated.

### 2.6 RLSP calculation validation with commercial phantoms

To validate the accuracy of the RLSP calculations, the calculations were also verified using commercial phantoms. Six tissue‐substitute inserts from a GAMMEX 467 phantom (Middleton, WI) were used: lung LN‐450 (model 485), adipose AP6 (model 453), CT solid water (SW) (model 451), liver LV1 (model 482), CB2‐30% CaCO_3_ (model 484) and cortical bone SB3 (model 450). The PDs and ECs provided by the manufacturer were used to calculate their RLSPs. These calculated RLSPs were then compared with previous RLSP measurements performed by institutional colleagues using the same accelerator facility and same Peakfinder device.^30^ Those reference measurements were obtained using a 163.3 MeV/n proton beam and a 312.7 MeV/n carbon ion beam. The six inserts were scanned using the same CT protocol described in Section 2.2 for animal tissues to obtain DEI values.

### 2.7 Sensitivity of CTN and RLSP on EC

To analyze the sensitivity of CTN to EC, the method of Schneider et al.^7^ that reformulated an equation by Rutherford et al ^18^ was used. This equation, expressed in CTN, is given in Equation (5).

(5) $$CTN=1000+RED\;\times \;\left(K^{ph}{\tilde{Z}}^{3.62}+K^{coh}{\hat{Z}}^{1.86}+K^{KN}\right)$$

where K^ph^, K^coh^, and K^KN^ are locally fitted coefficients representing the photoelectric interaction, coherent scattering and Compton scattering, respectively. $\tilde{Z}$ and $\hat{Z}$ are effective numbers of the test samples. In this study, the fitting coefficients were first determined using several of the tissue substitute materials in the GAMMEX 467 phantom: SW, LV1, and SB3. The effective atomic numbers of each animal tissue were then calculated from the laboratory‐measured ECs.

Using this approach, the effect of EC variations on both CTN and RLSP was investigated by varying selected elemental fractions in two representative tissues: Kidney (soft tissue) and mandible (bone). For each trial variation in the animal tissues, only two elements were altered at the same time. For kidney, the mass fraction of the first element was increased by 5% from the corresponding laboratory‐measured value, while that of the second was decreased by 5%, keeping the total sum of all components at 100%. For mandible, variations of either 10% or 9% were used depending upon the elements involved. When P was involved, a 9% change was used because its original mass fraction was 9.5% and a larger decrease would lead to a negative value. Percentage change in CTN (120 kVp), RED, and RLSP resulting from elemental composition variations were then evaluated.

### 2.8 DECT‐based tissue characterization

For MC simulations that require PD per voxel, a lookup array was constructed to convert SCTN and DEI (scans at 140 kVp and 80 kVp) into PD. Data input into the PD array included the values from the 12 animal tissues from this study, 32 non‐tissue materials (see Appendix), and for SCTN between 0 and 1000, data points that assume a linear mixture of air and water. The mixture in this range of SCTN was used to account for partial‐volume voxels (e.g., voxels containing both soft tissue and air) which is a common occurrence. For SCTN values above 1000 (i.e., materials with PD greater than water), the PD array was populated solely with directly measured densities from the high‐density materials listed above, without any mixing assumption. The array spans SCTNs from 0 to 4095 (the maximum measured value was 2885 for aluminum) and DEIs multiplied by 10 000 to yield integer values from –300 to 1800. For clinical use, if a (CTN, DEI) pair lies outside the measured or interpolated ranges, the TPS prompts the user to verify and manually enter values; extrapolation is not permitted.

## 3 RESULTS

Table 2 lists for each sample the measured thickness, the mean single‐energy CT number (SCTN) at 120 kVp with its standard deviation (SD) over the region of interest, and the measured RLSPs for proton and carbon ion beams at the indicated energies. It shows that the measured RLSPs for the two ion species and three beam energies were nearly identical (within ± 1%), except for lung, which showed a maximum difference of nearly 2%.

**TABLE 2:** Sample name, thickness (mm), SCTN (120 kVp) ± SD, and measured RLSPs for proton and carbon ion beams using different energies.

			Proton (MeV/n)			Carbon ion (MeV/n)		
sample name	Thickness (mm)	120 kVp SCTNs ± SD_ROI_	221.1	150.1	104.5	430.1	285.4	195.7
lung	24.90	400 ± 106	0.476	0.473	0.478	0.470	0.464	0.468
adipose	24.90	919 ± 2	0.975	0.981	0.981	0.981	0.979	0.979
water	25.00	1000 ± 3	1.000	1.000	1.000	1.000	1.000	1.000
vitreous	29.90	1007 ± 7	1.006	1.007	1.005	1.006	1.004	1.007
brain	24.90	1037 ± 4	1.034	1.034	1.034	1.035	1.035	1.036
kidney	24.90	1052 ± 3	1.036	1.041	1.034	1.035	1.040	1.036
liver	24.90	1063 ± 4	1.048	1.053	1.054	1.049	1.051	1.055
muscle	24.90	1064 ± 4	1.054	1.058	1.055	1.052	1.054	1.056
heart	24.90	1055 ± 4	1.056	1.058	1.056	1.056	1.055	1.056
cartilage	29.10	1090 ± 8	1.057	1.058	1.059	1.058	1.058	1.058
scapula	11.10	1277 ± 126	1.153	1.153	1.153	1.153	1.152	1.153
mandible	13.50	1742 ± 399	1.326	1.328	1.324	1.326	1.325	1.323
teeth	28.10	2870 ± 562	1.822	1.818	1.821	1.818	1.818	1.820

Figure 3 plots the RLSPs for all six ion beams versus the measured single‐energy (120 kVp) SCTNs for the 12 animal tissue samples. Figure 3 also plots a curve presented in reference 9 that was used for comparing conversion functions used at 10 proton treatment facilities in the U.S. (some used multiple scanners or protocols).^9^ For soft tissues, measured RLSPs differed from the standard‐set RLSPs by less than 2%. For the high‐density samples (scapula, mandible, teeth), the measured RLSP curve lies below and runs parallel to the standard curve. The scapula sample was only half as thick as the others, making its RLSP measurement less reliable. The tooth sample showed a −5.3% difference between the measured and standard conversion function RLSPs.

**FIGURE 3:**
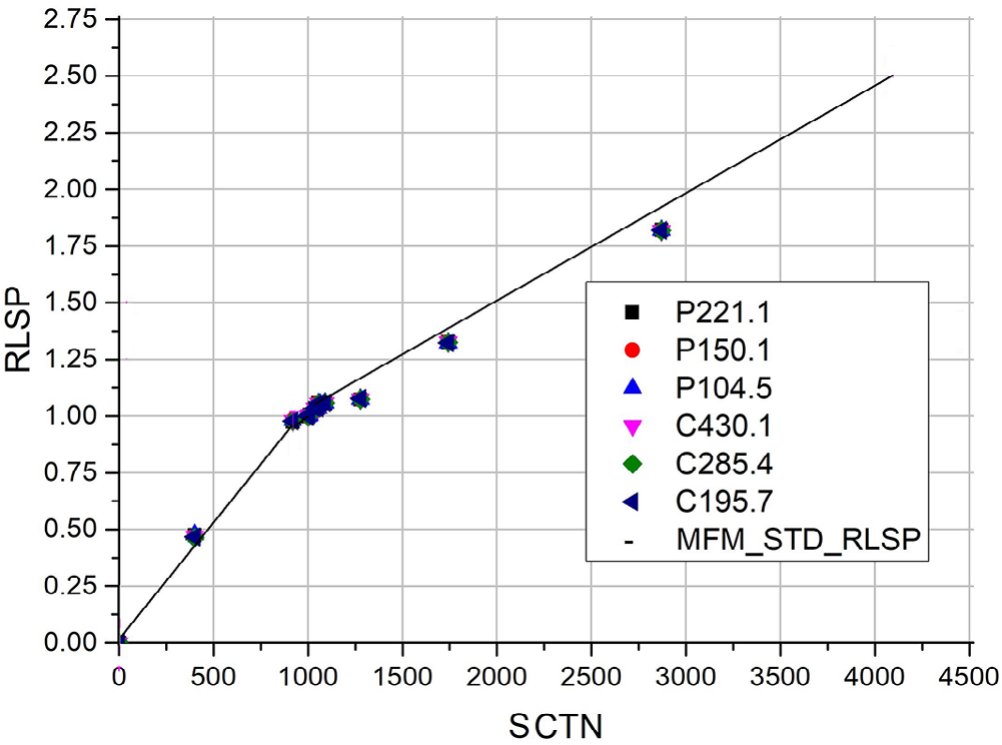
Measured RLSPs versus SCTNs at 120 kVp for the six proton and carbon ion beams. The legend identifies the ion species and energy per nucleon. A standard SCTN‐to‐RLSP conversion curve for 120 kVp is also shown (solid line).^9^

Table 3 lists for each tissue sample the measured PD (columns 2), the literature PD (columns 3) from ICRU 44,^31^ the percentage difference between them (columns 4), the measured SCTN at 140 kVp, the RED, and the DEI values. The differences between measured and literature physical densities were less than ± 1.6 % for all soft tissues except lung. For higher‐density samples, the literature densities deviated from measured densities by as much as 32.4 %.

**TABLE 3:** Measured and literature PDs, difference (%), SCTN at 140 kVp, RED, and DEI for each animal tissue sample.

sample	PD_meas_ (g/cm^3^)	PD_Lit_(g/cm^3^)	Diff PD_Lit_to PD_meas_(%)	140 kVp SCTNs ± SD_ROI_	RED	DEI
lung	0.457 ± 0.69%	0.26	−43.11	411 ± 110	0.456	0.007
adipose	0.950 ± 0.05%	0.95	0.00	926 ± 3	0.947	−0.015
vitreous	1.004 ± 0.09%	—	—	1000 ± 3	1.000	0.001
brain	1.036 ± 0.24%	1.04	0.39	1004 ± 8	1.014	0.002
kidney	1.048 ± 0.05%	1.05	0.19	1033 ± 4	1.023	0.001
liver	1.051 ± 0.12%	1.06	0.86	1034 ± 4	1.037	0.001
muscle	1.052 ± 0.06%	1.05	−0.19	1063 ± 4	1.052	0.002
heart	1.068 ± 0.05%	1.06	−0.75	1059 ± 5	1.044	0.005
cartilage	1.083 ± 0.12%	1.10	1.57	1054 ± 5	1.047	0.003
scapula	1.172 ± 1.32%	1.29	10.06	1091 ± 9	1.052	0.001
mandible	1.513 ± 1.25%	1.68	11.04	1251 ± 130	1.159	0.083
teeth	2.077 ± 2.05%	2.75	32.40	1690 ± 400	1.40	0.085

The EC results from laboratory measurements of the twelve animal tissue samples are presented in Table 4. For comparison, literature ECs of human tissues from Woodard and White^20^ are also listed. Since their tabulation does not provide data for scapula, data for sacrum was instead used for the comparison. Likewise, vitreous humor data was not given in their report; however, vitreous humor is approximately 97% water,^32^ so no EC tissue data is provided in table 4 for comparison. The EC data for the six GAMMEX 467 tissue‐substitute inserts, supplied by the manufacturer, are also listed in Table 4.

**TABLE 4:** Elemental composition (mass %) of twelve animal tissues (laboratory analysis, top row) compared with reference data from Woodard and White^20^ and six GAMMEX 467 inserts (bottom row of each tissue).

sample name	H	C	N	O	Na	Mg	P	S	Cl	K	Ca	Fe	Ar	Si
air	0.00	0.00	75.5	23.20	0.00	0.00	0.00	0.00	0.00	0.00	0.00	0.00	1.30	0.00
lung^+^	11.03	13.29	3.10	72.001	0.02	0.01	0.15	0.11	0.11	0.10	0.07	0.00	0.00	0.00
inflated lung～	10.300	10.50	3.10	74.90	0.20	0.00	0.20	0.30	0.30	0.20	0.00	0.00	0.00	0.00
LN‐450*	8.50	59.60	2.00	18.10	0.00	11.20	0.00	0.00	0.10	0.00	0.00	0.00	0.00	0.60
adipose^+^	10.99	68.38	0.32	20.24	0.00	0.00	0.00	0.00	0.07	0.00	0.00	0.00	0.00	0.00
adipose～	11.40	59.80	0.70	27.80	0.10	0.00	0.00	0.10	0.10	0.00	0.00	0.00	0.00	0.00
AP6*	9.06	72.30	2.25	16.27	0.00	0.00	0.00	0.00	0.13	0.00	0.00	0.00	0.00	0.00
vitreous^+^	12.37	2.88	1.11	82.75	0.21	0.00	0.01	0.24	0.39	0.03	0.00	0.00	0.00	0.00
water	11.19	0.00	0.00	88.81	0.00	0.00	0.00	0.00	0.00	0.00	0.00	0.00	0.00	0.00
SW 451*	8.00	67.30	2.39	19.87	0.00	0.00	0.00	0.00	0.14	0.00	2.31	0.00	0.00	0.00
brain^+^	9.85	11.78	3.50	74.10	0.05	0.02	0.25	0.10	0.01	0.25	0.09	0.00	0.00	0.00
brain～	10.70	14.50	2.20	71.20	0.20	0.00	0.40	0.20	0.30	0.30	0.00	0.00	0.00	0.00
kidney^+^	10.13	11.31	2.77	73.46	1.26	0.16	0.37	0.01	0.29	0.22	0.01	0.01	0.00	0.00
kidney～	10.30	13.20	3.00	72.40	0.200	0.00	0.20	0.20	0.20	0.20	0.10	0.00	0.00	0.00
liver^+^	11.33	14.03	3.39	69.06	0.55	0.08	0.24	0.01	0.19	1.10	0.01	0.01	0.00	0.00
liver～	10.20	13.90	3.00	71.60	0.20	0.00	0.30	0.30	0.20	0.30	0.00	0.00	0.00	0.00
LV1*	8.06	67.01	2.47	20.01	0.00	0.00	0.00	0.00	0.14	0.00	2.31	0.00	0.00	0.00
muscle^+^	10.38	14.32	3.83	69.43	0.20	0.11	0.14	0.00	0.12	1.47	0.00	0.00	0.00	0.00
muscle～	10.20	14.30	3.40	71.00	0.10	0.00	0.20	0.30	0.10	0.40	0.00	0.00	0.00	0.00
heart^+^	9.14	11.40	3.16	76.12	0.00	0.00	0.00	0.04	0.14	0.00	0.00	0.00	0.00	0.00
heart～	10.3	12.10	3.20	73.40	0.10	0.00	0.10	0.20	0.30	0.20	0.00	0.10	0.00	0.00
cartilage^+^	8.01	14.53	4.56	72.51	0.00	0.00	0.00	0.00	0.28	0.10	0.01	0.00	0.00	0.00
cartilage～	9.60	9.90	2.20	74.40	0.50	0.00	2.20	0.90	0.30	0.00	0.00	0.00	0.00	0.00
CB2‐30%*	6.68	53.48	2.12	25.61	0.00	0.00	3.24	0.00	0.11	0.00	8.87	0.00	0.00	0.00
scapula^+^	10.12	10.99	3.41	62.67	0.26	0.14	4.78	0.80	0.02	0.05	6.78	0.01	0.00	0.00
sacrum～	7.40	30.20	3.70	43.80	0.00	0.10	4.50	0.20	0.10	0.10	9.80	0.10	0.00	0.00
mandible^+^	4.25	11.89	3.90	52.30	0.60	0.40	9.50	0.03	0.06	0.07	16.902	0.08	0.00	0.00
mandible～	4.60	19.90	4.10	43.50	0.10	0.20	8.60	0.30	0.00	0.00	18.70	0.00	0.00	0.00
SB3*	3.41	31.410	1.84	36.50	0.00	0.00	0.00	0.00	0.04	0.00	26.81	0.00	0.00	0.00
teeth^+^	4.04	10.500	2.87	50.50	0.78	0.46	11.206	0.02	0.05	0.04	19.31	0.18	0.00	0.00
teeth(enamel) ～ ＾	2.20	9.50	2.90	42.10	0.00	0.70	13.70	0.00	0.00	0.00	28.90	0.00	0.00	0.00

^+^ ≡EC data determined by laboratory analysis, ～ ≡EC data from Woodard and White,^20^ *≡substitute phantom material.

Table 5 lists and compares the RLSP calculations to measurements. Since proton and carbon ion measured RLSPs are nearly identical at multiple energies, only the data for the proton energy of 104.5 MeV/n, which shows the largest differences between calculated and measured values, is given. The table lists the RLSPs calculated by three different combinations of EC and PD and the differences between each calculated value and the measured value given in table 2. The three combinations are: laboratory EC with measured PD (column 2), literature EC with literature PD (column 3), and literature EC with measured PD (column 4). The corresponding differences from the measured RLSPs are shown in columns 5, 6, and 7, respectively.

**TABLE 5:** Measured and calculated RLSPs for 104.5 MeV/n protons using three different calculation methods, with the respective differences.

sample	RLSP EC_Lab_ and PD_meas_	RLSP EC_Lit_ and PD_lit_	RLSP EC_Lit_ and PD_meas_	ΔRLSP using PD_meas_ (%)	ΔRLSP using PD_Lit_ (%)	ΔRLSP using PD_meas_ (%)
lung	0.458	0.258	0.453	−4.27	−46.09	−5.24
adipose	0.964	0.966	0.966	−1.74	−1.49	−1.49
vitreous	1.019	—	—	1.39	—	—
brain	1.022	1.036	1.033	−1.17	0.24	−0.14
kidney	1.010	1.041	1.039	0.35	0.72	0.53
liver	1.055	1.050	1.041	0.08	−0.38	−1.22
muscle	1.044	1.041	1.043	−1.06	−1.37	−1.18
heart	1.045	1.051	1.059	−1.08	−0.47	0.28
cartilage	1.045	1.078	1.061	−1.31	1.80	0.23
scapula	1.148	1.248^*^	1.170	−0.45	8.28	1.62
mandible	1.341	1.515	1.365	1.31	14.44	3.06
teeth	1.846	2.424^+^	1.831	1.36	33.12	0.54

* ≡ sacrum, + ≡ enamel, PD_meas_ ≡ PDs measured in this study. PD_Lit_ ≡ PDs taken from ICRU 44 report. Ele_Lab_ ≡ ECs measured by laboratory, Ele_Lit_ ≡ ECs taken from literature. Lab_Calc_ ≡ RLSPs calculated using Laboratory EC, Lit_Calc_ ≡ RLSPs calculated using literature EC.

When laboratory measured EC and measured PD were used (column 5), the maximum difference was −4.27% and the average difference was −0.55% ± 1.62% (1 SD). In contrast, the RLSPs from literature EC and literature PD (column 6) showed much larger differences, some as large as −46% and + 33%. When literature EC was combined with measured PD (column 7), the differences dropped to between −5.3% and + 3.1%, with an average difference of −0.27% ± 2.11% (1 SD). In summary, the best agreement between calculated and measured RLSPs is achieved using measured PD with measured EC, followed by measured PD with literature EC, while the worst agreement occurs when both PD and EC are taken from the literature.

The validation of the RLSP calculation method using the inserts from a commercial phantom is presented in Table 6, together with results for the real animal lung tissue. For lung tissue, both the inflated and deflated states share the same elemental composition; only the volume of air affects the PD. In this study, the uncompressed animal lung (PD  =  0.457 g/cm^3^) had a PD substantially different from the inflated (0.26 g/cm^3^) and deflated (1.05 g/cm^3^) literature values.^31^ The real lung tissue showed proton and carbon ion ΔRLSP of –4.27% and –2.62%, respectively, while the GAMMEX LN 450 phantom exhibited larger discrepancies of –4.66% and –6.13%. Among the remaining GAMMEX inserts (excluding LN 450), all measured RLSPs agreed with calculated values to within ± 2.1% (Table 6), similar to the accuracy observed for animal tissues (Table 5).

**TABLE 6:** Comparison of calculated and measured RLSPs for GAMMEX 467 inserts^30^ and for real lung tissue (this study), for proton and carbon ions.

material	PD(g/cm^3^)	DEI	measured RLSP(P)	calculated RLSP(P)	ΔRLSP (%)(P)	measured RLSP(C)	calculated RLSP(C)	ΔRLSP (%)(C)
Lung^+^	0.457	0.007	0.478	0.4586	−4.27	0.470	0.458	−2.62
LN‐450(485)*	0.450	0.004	0.46	0.4386	−4.66	0.467	0.438	−6.13
AP6*(453)	0.940	−0.013	0.952	0.9427	−0.98	0.956	0.944	−1.27
SW‐451^*^(451)	1.020	0.005	1.008	0.9958	−1.21	1.014	0.995	−1.89
LV1^*^(482)	1.100	0.002	1.085	1.0647	−1.87	1.088	1.065	−2.10
CB2‐30%^*^(484)	1.340	0.057	1.272	1.2620	−0.79	1.275	1.259	−1.26
SB3^*^(450)	1.820	0.113	1.624	1.6225	−0.09	1.629	1.610	−1.15

P: proton, C: carbon, LN‐450: lung ‐450, CB2‐30%: CB2‐30% CaCO3, + ≡ sample in this study, *≡substitute phantom material.

Table 4 shows that the most abundant elements in soft tissue and cartilage are H, O, and C, while in mandible and teeth the most abundant are P, O, and Ca. The effect of EC variations on calculated RLSPs was investigated for kidney and mandible using the method described in Section 2.7. Table 7 presents the trial elemental concentration matrix and the resulting percentage changes in RLSPs. In the table, an example entry of “O‐5/Ca+5” means that oxygen was decreased by 5% and calcium increased by 5% relative to their original percentages. The trial calculations illustrate that the tested percentage changes in C, O, Ca, and P for high‐density materials caused less than ± 1% differences in calculated RLSPs. On the other hand, even small variations in H concentration in soft tissues (by as little as 5%) can have a large impact on RLSP accuracy. The percentage changes in CTN with the various test changes of EC all resulted in less than 0.2 % difference.

**TABLE 7:** Percentage changes in CTN, RED, and RLSP resulting from elemental composition variations in kidney and mandible, relative to laboratory‐determined ECs.

items	EC variation in percentage					
kidney	O‐5/C+5	O‐5/H+5	H‐5/O+5	H‐5/C+5	C‐5/O+5	C‐5/H+5
CTN (%)	0.079	0.078	0.077	−0.077	−0.079	0.077
RED diff (%)	0.29	4.20	4.73	−4.74	0.26	3.91
ΔRLSP (%)	0.14	6.60	−6.62	−6.46	−0.16	6.46
mandible	Ca‐10/P+10	Ca‐10/O+10	P‐9/Ca+9	P‐9/O+9	O‐10/Ca+10	O‐10/P+10
CTN (%)	0.152	0.144	0.154	0.162	0.144	0.161
RED diff (%)	0.28	0.00	0.21	0.28	0.00	0.35
ΔRLSP (%)	−0.08	0.91	0.11	0.91	−0.90	−0.99

As an illustration of the DECT‐based lookup approach (described in Section 2.8), the measured (SDECTN, DEI) pairs at 140 kVp were used to generate the PD array shown in Figure 4. The PD array can be considered an image with a pixel value depth of 2^12^ thereby supporting a PD resolution better than 0.1% up to a maximum value of 4.095 g/cm^3^, greater than any natural tissue in the body. Some implanted objects and set‐up equipment have PDs greater than this value but most CT scanners only provide a maximum CTN of 4095. With this image scheme, when converting [SCTN, DEI] pairs to [PD] pairs, no interpolation is needed and the converted values are acquired simply and fast by indexing the image.

**FIGURE 4:**
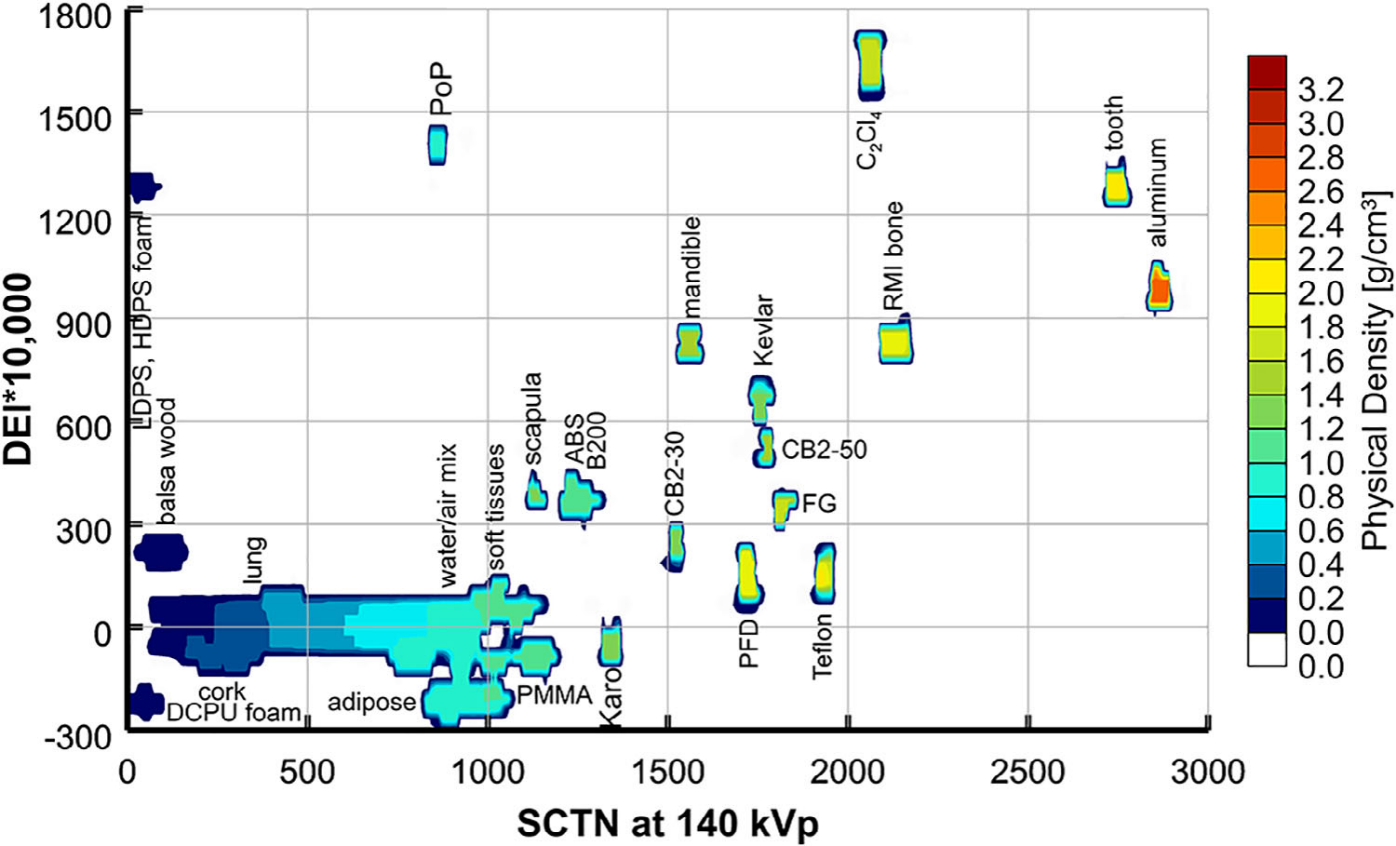
PD as a function of SCTN and DEI (140 kVp and 80 kVp). Color bar = PD; white = no data. The SCTN values in the array extend from 0 to 4095 but the largest SCTN measured in this study was only 2885 so this graph only includes data for SCTN up to 3000. For better visualization of the physical density values, a small region around each data point is colored according to the density values.

## 4 DISCUSSION

When using Monte Carlo calculations that rely on voxel‐level PD, measured densities are preferred over calculated ones, because the density of liquids and solids depends on molecular packing or crystal structure. As shown in Table 5, the best agreement between measured and calculated RLSP is obtained when measured densities are used, even if literature EC substitutes for measured EC. Most published tissue ECs are derived from fundamental components (water, lipid, protein, etc.) and may vary with age, gender, or health condition.^19^, ^33^ CTN‐to‐RLSP conversion functions are typically derived from bulk tissue measurements, yielding weighted averages of components such as water, fat, and bone. For scanning spot beams and MC simulations, however, such bulk averages may not accurately represent local interactions. By using small tissue samples and a narrow beam, this study reduces the uncertainties associated with bulk averaging.

Literature on measured RLSP of teeth is sparse due to their irregular shape, heterogeneous composition (enamel, dentin, pulp), and small size. The outermost enamel is highly mineralized (≈ 96% hydroxyapatite) with reported densities of 2.75–3.0 g/cm^3^.^34^ The middle region dentin, with a typical composition of approximately 12% water, 18% organic matrix, and 70% inorganic matter by weight, typically has a density between 1.7 to 2.1 g/cm^3^.^35^ The innermost region is the pulp, which contains the blood supply, nerves, and other soft tissues and has an expected density of 1.0 to 1.1 g/cm^3^.^36^ In this study, fresh cow teeth (containing organic matrix and residual water) were used to represent in vivo conditions. Whole teeth were not, however, separated into distinct regions; the measured average PD of 2.077 g/cm^3^ therefore reflects a volume‐weighted combination of pulp, dentin, and enamel. For stability during RLSP measurements, incisor and molar teeth were placed together with the beam passing primarily through the molars. Although the calcium concentration differed by about 9% from values reported by Woodard and White,^20^ simulations confirmed that small changes in the Ca fraction does not significantly affect the calculated RLSP (less than ± 1%). This evaluation directly assesses the RLSP derived from the specified elemental composition.

This study highlights that direct RLSP calculation from accurate EC and measured PD are more reliable for bone. PD and H concentration are the main factors affecting calculated RLSPs, consistent with the findings of Yang et al.^15^ For kidney (soft tissue), a 5% variation in H caused RLSP changes up to 6.6%. The maximum changes in RLSP for kidney were observed when the H concentration was varied together with either C or O, but not when the C and O were exchanged. For mandible (bone), a 10% variation in Ca and O altered the RLSP less than 1%. For head and neck treatments, proton beams typically traverse less than 20 mm of teeth. The 5.3% RLSP difference observed for teeth would translate into a range error of approximately 1 mm, which is well within typical clinical PTV margins of 3 to 5 mm.^5^ The uncertainty introduced by using literature values for teeth is therefore likely clinically acceptable. In contrast, beam traversal through thicker bones such as the mandible, scapula, or pelvis may result in larger errors, as suggested by the up to 32% PD difference in literature densities for high‐density samples, which should be considered in treatment planning. Literature data indicate that the difference between the PD of bovine bone (≈ 1.96 ± 0.08 g/cm^3^ ^37^) and human cortical bone (1.822 g/cm^3^) ^31^ is much smaller than the observed difference in this study suggesting that the observed difference is primarily due to the difference between fresh, hydrated tissue and fully mineralized reference data rather than a species‐specific effect.

A limitation of this study is the indirect volume measurement method (water displacement), which may introduce small errors in the density determination for irregular samples. Future work could employ dental cone beam CT or beams with small‐apertures which could better limit the CTN and RLSP measurements to individual tooth regions. The superior spatial resolution of CBCT (with voxel sizes between 0.1 and 0.4 mm could allow for a more precise delineation of enamel, dentin, and pulp, thereby improving the accuracy of path length and composition estimates for RLSP calculations.

The lung sample showed the largest difference between measured and calculated RLSP, consistent with the LN‐450 substitute ^38^ and with Witt et al.^39^ Yang et al. ^40^ reported a 3.7% CTN uncertainty for lung, leading to ≈ 3.8% RLSP uncertainty; combined uncertainties could reach ≈ 5%. The difference for real lung was – 4.27% for 104.5 MeV protons and –2.62% for 430.1 MeV/n carbon ions.

Due to limited beam time, each RLSP measurement was repeated three times without removing and repositioning the samples between repeats. This approach avoided additional dehydration or mechanical damage to the fresh animal tissues, but it did not account for setup uncertainties (like repositioning or sample orientation). As a result, the reported standard differences reflect only the short‐term repeatability under fixed geometry, not the full inter‐measurement variability that would include reinstallation of the specimens. The use of animal tissues, with their inherent inhomogeneity and susceptibility to change, further limits the direct extrapolation of these uncertainty estimates to clinical practice. Despite these limitations, the consistent pattern observed across tissues supports the conclusion that measured PD and measured EC yield the closest agreement with RLSP, while literature PD and literature EC produce the largest discrepancies. This is consistent with the expectation that measured values reflect the specific composition and hydration state of the fresh animal tissues used here, whereas literature reference values typically derive from different tissue sources or preparation methods (fixed or dried samples). Given that PD is the most critical factor in RLSP calculation, improving the CTN‐to‐PD conversion function will directly enhance the accuracy of calculated RLSPs.

The present study supports the feasibility of converting SECT and DECT images into PD, EC, and RLSP arrays for Monte Carlo calculations. An advantage of DECT compared to SECT is its ability to reduce material mis‐assignment.^41^ One example from this study is for two materials with nearly the same SCTN; Teflon (C_2_F_4_) and tetrachloroethylene (C_2_Cl_4_) that both have SCTNs near that of bone (≈ 2000) but have very different PDs of 2.18 and 1.60 g/cm^3^ respectively. The DEI parameter effectively distinguishes these materials.  In another common scenario, DEI can differentiate a voxel containing a muscle‐air mixture from one containing only adipose. Voxels with water or most soft tissues typically show DEIs ranging from –0.02 to + 0.03, whereas adipose exhibits DEIs near –0.07. It should be noted that even with DECT, a few materials with nearly identical (CTN, DEI) pairs showed PD differences up to 2.4%; nevertheless, this represents an improvement over SECT‐only methods, which have a 3 to 4% uncertainty at the 2 SD level.

To the authors' knowledge, this is the first study since the 1970s to measure PD, EC, single‐energy CTN, dual‐energy CTN, and proton‐ and carbon‐ion measured RLSP on identical tissue samples, and the first to report such data for real teeth. Modern elemental analysis techniques now enable updated validation of CTN‐to‐RLSP calibration functions for ion beam therapy.

## 5 CONCLUSIONS

In this study, the measured RLSPs for 12 animal tissues showed no significant dependence on beam energy or ion species. For all tissues except lung (within 4.3%), the agreement between measured RLSPs and those calculated from laboratory‐ determined EC and measured PD was within 2%.

Accurate RLSP calculations depend primarily on PD and hydrogen content. DECT offers advantages over SECT in resolving different materials with the same CTN. A new image‐based scheme has been proposed to convert DECT images to maps of material properties which simplifies the use of DEXCT for MC based planning and reduce the planner's effort for manual contouring and assigning materials.

## AUTHOR CONTRIBUTIONS

Xiaoli Liu and Michael Moyers designed the study. Xiaoli Liu performed the measurements, analyzed the data, drafted and revised the manuscript. Michael Moyers verified the results and contributed to manuscript revision. All authors read and approved the final manuscript.

## CONFLICT OF INTEREST STATEMENT

The authors have no relevant conflicts of interest to disclose.

## ETHICAL STATEMENT

Ethical approval was not required for this study.

## FUNDING INFORMATION

A hospital funding from shanghai proton and heavy ion center, which is SPHIC rising star.

## Data Availability

The data that support the findings of this study are available from the corresponding author upon reasonable request.

## 1

As described in the text, Monte Carlo simulations require PD and EC, whereas initial portal optimization still needs RLSP to select beam energies. In addition to the 12 tissues investigated in this study, several other tissue and non‐tissue materials are also required for treatment planning and are included in Figure 4. Table A.1 lists the materials entered into the Monte Carlo‐based TPS database for treatment planning.

**TABLE A.1:** Materials entered into the Monte Carlo‐based TPS and shown in Figures 4. Materials are ordered first by increasing CTN, then by increasing DEI.

#	material	measured density [g/cm^3^]	#	material	measured density [g/cm^3^]
1	LDPS foam	0.015	17	PU	1.012
2	HDPS foam	0.030	18	blue SW (HE 557)	1.035
3	DCPU foam	0.038	19	red SW (457)	1.043
4	balsa wood	0.115	20	bolus ABS	1.125
5	cork	0.240	21	B200	1.148
6	TPL lung	0.324	22	PMMA	1.185
7	RMI lung	0.342	23	CB2‐30	1.316
8	95% ethyl alcohol	0.811	24	Kevlar composite	1.329
9	Plaster of Paris	0.822	25	Karo syrup	1.397
10	paraffin wax	0.900	26	CB2‐50	1.551
11	bolus wax	0.926	27	C_2_Cl_4_	1.675
12	sunflower oil	0.930	28	FG composite	1.700
13	pod PE	0.942	29	RMI bone	1.820
14	HDPE	0.964	30	PFD	1.979
15	TPL tissue	1.011	31	Teflon	2.179
16	Spectra composite	1.012	32	aluminum	2.682
